# The genome sequence of the flounced rustic, *Luperina testacea *(Denis & Schiffermüller, 1775)

**DOI:** 10.12688/wellcomeopenres.17816.1

**Published:** 2022-04-05

**Authors:** Gavin R. Broad, Natural History Museum Genome Acquisition Lab, Darwin Tree of Life Barcoding collective, Wellcome Sanger Institute Tree of Life programme, Wellcome Sanger Institute Scientific Operations: DNA Pipelines collective, Tree of Life Core Informatics collective, Darwin Tree of Life Consortium

**Affiliations:** 1 Department of Life Sciences, Natural History Museum, London, UK

**Keywords:** Luperina testacea, flounced rustic, genome sequence, chromosomal, Lepidoptera

## Abstract

We present a genome assembly from an individual male *Luperina testacea* (the flounced rustic; Arthropoda; Insecta; Lepidoptera; Noctuidae). The genome sequence is 601 megabases in span. The majority of the assembly (99.98%) is scaffolded into 31 chromosomal pseudomolecules, with the Z sex chromosome assembled. The mitochondrial genome was also assembled, and is 15.3 kilobases in length.

## Species taxonomy

Eukaryota; Metazoa; Ecdysozoa; Arthropoda; Hexapoda; Insecta; Pterygota; Neoptera; Endopterygota; Lepidoptera; Glossata; Ditrysia; Noctuoidea; Noctuidae; Noctuinae; Apameini; Luperina; *Luperina testacea* (Denis & Schiffermüller, 1775) (NCBI:txid988002).

## Background

Across much of north-west and central Europe, Luperina testacea, the flounced rustic, is a common moth of late summer and autumn. Widespread in Britain, Luperina testacea becomes much less frequent in Scotland. Eggs are laid at the bases of various grasses and the smooth, rather unpigmented larvae feed on the roots and lower stems, feeding slowly through the winter. Adults do not feed, are regular at light (predominantly males) and can be one of the most abundantly trapped moths from late July to early October, especially in open coastal areas or calcareous grasslands ( Henwood *et al.*, 2020; Waring *et al.*, 2003). Abundance in southern Sweden is positively associated with soil disturbance ( Tyler, 2020).

Adults are variable in appearance, particularly in the tone of the forewing. The hind wings are always strikingly pale, the fore wings usually have a distinct dark bar in the centre and a broad, paler subterminal area. 

## Genome sequence report

**Figure 1.**
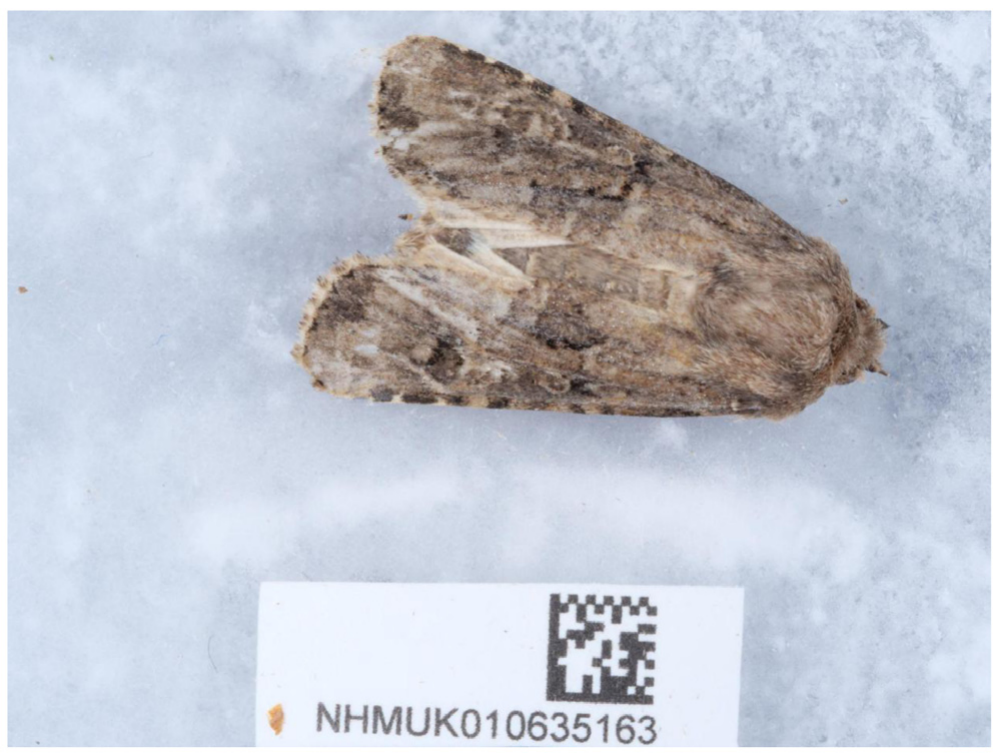
Image of the *Luperina testacea* (ilLupTest1) specimen taken prior to preservation and processing.

The genome was sequenced from one male *L. testacea* ( Figure 1) collected from Hever Castle, England, UK (latitude 51.1884, longitude 0.1198). A total of 26-fold coverage in Pacific Biosciences single-molecule long reads and 76-fold coverage in 10X Genomics read clouds were generated. Primary assembly contigs were scaffolded with chromosome conformation Hi-C data. Manual assembly curation corrected 24 missing/misjoins, reducing the assembly size by 0.01% and the scaffold number by 33.33%, and increasing the scaffold N50 by 4.57%.

**Figure 2.**
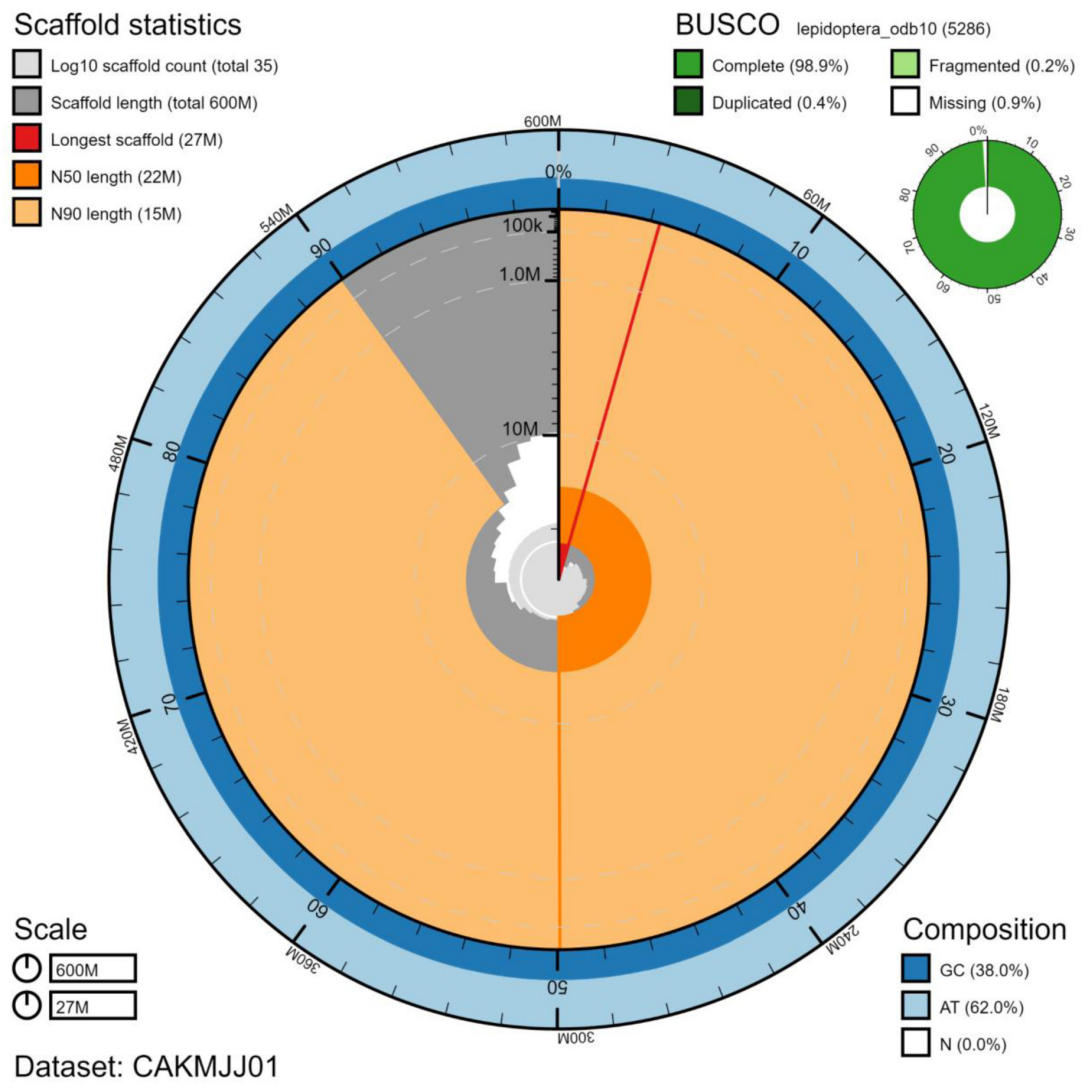
Genome assembly of *Luperina testacea*, ilLupTest1.1: metrics. The BlobToolKit Snailplot shows N50 metrics and BUSCO gene completeness. The main plot is divided into 1,000 size-ordered bins around the circumference with each bin representing 0.1% of the 601,512,407 bp assembly. The distribution of chromosome lengths is shown in dark grey with the plot radius scaled to the longest chromosome present in the assembly (26,814,763 bp, shown in red). Orange and pale-orange arcs show the N50 and N90 chromosome lengths (21,613,865 and 15,062,659 bp), respectively. The pale grey spiral shows the cumulative chromosome count on a log scale with white scale lines showing successive orders of magnitude. The blue and pale-blue area around the outside of the plot shows the distribution of GC, AT and N percentages in the same bins as the inner plot. A summary of complete, fragmented, duplicated and missing BUSCO genes in the lepidoptera_odb10 set is shown in the top right. An interactive version of this figure is available at https://blobtoolkit.genomehubs.org/view/ilLupTest1.1/dataset/CAKMJJ01/snail.

The final assembly has a total length of 601 Mb in 34 sequence scaffolds with a scaffold N50 of 21.6 Mb ( Table 1). The majority of the assembly sequence (99.98%) was assigned to 31 chromosomal-level scaffolds, representing 30 autosomes (numbered by sequence length), and the Z sex chromosome ( Figure 2– Figure 5; Table 2). The assembly has a BUSCO v5.2.2 ( Manni *et al.*, 2021) completeness of 98.9% (single 98.6%, duplicated 0.4%) using the lepidoptera_odb10 reference set. While not fully phased, the assembly deposited is of one haplotype. Contigs corresponding to the second haplotype have also been deposited.

**Table 1.** Genome data for *Luperina testacea*, ilLupTest1.1.

*Project accession data*	
Assembly identifier	ilLupTest1.1
Species	*Luperina testacea*
Specimen	ilLupTest1
NCBI taxonomy ID	NCBI:txid988174
BioProject	PRJEB48331
BioSample ID	SAMEA8534287
Isolate information	Male, thorax (genome assembly), head (Hi-C)
*Raw data accessions*	
PacificBiosciences SEQUEL II	ERR7221643
10X Genomics Illumina	ERR7220448-ERR7220451
Hi-C Illumina	ERR7220452
*Genome assembly*	
Assembly accession	GCA_927399505.1
Accession of alternate haplotype	GCA_927399495.1
Span (Mb)	601
Number of contigs	68
Contig N50 length (Mb)	17.1
Number of scaffolds	34
Scaffold N50 length (Mb)	21.6
Longest scaffold (Mb)	24.7
BUSCO * genome score	C:98.9%[S:98.6%,D:0.4%], F:0.2%,M:0.9%,n:5286

*BUSCO scores based on the lepidoptera_odb10 BUSCO set using v5.2.2. C= complete [S= single copy, D=duplicated], F=fragmented, M=missing, n=number of orthologues in comparison. A full set of BUSCO scores is available at https://blobtoolkit.genomehubs.org/view/ilLupTest1.1/dataset/CAKMJJ01/busco.

**Figure 3.**
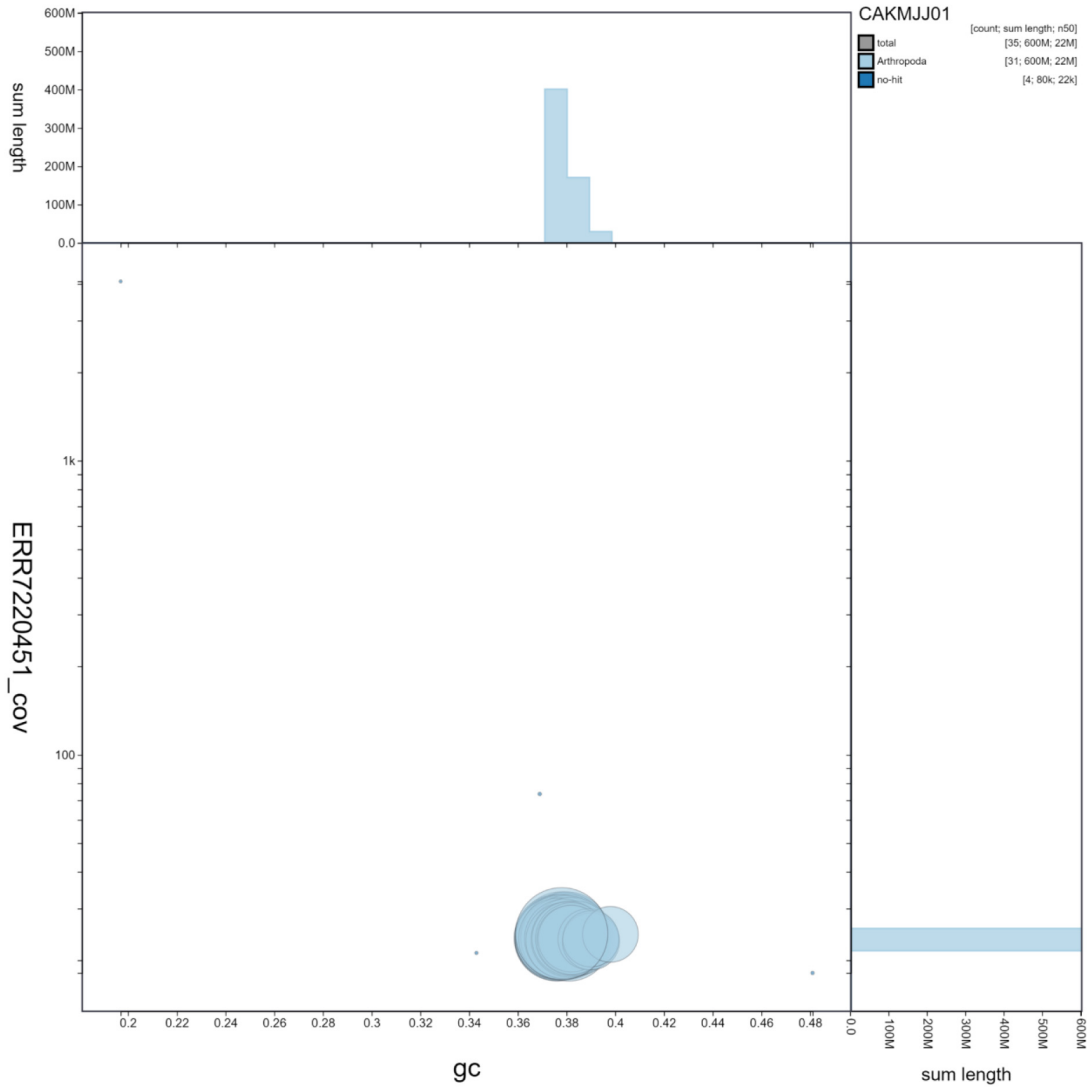
Genome assembly of *Luperina testacea*, ilLupTest1.1: GC coverage. BlobToolKit GC-coverage plot. Scaffolds are coloured by phylum. Circles are sized in proportion to scaffold length Histograms show the distribution of scaffold length sum along each axis. An interactive version of this figure is available at https://blobtoolkit.genomehubs.org/view/ilLupTest1.1/dataset/CAKMJJ01/blob.

**Figure 4.**
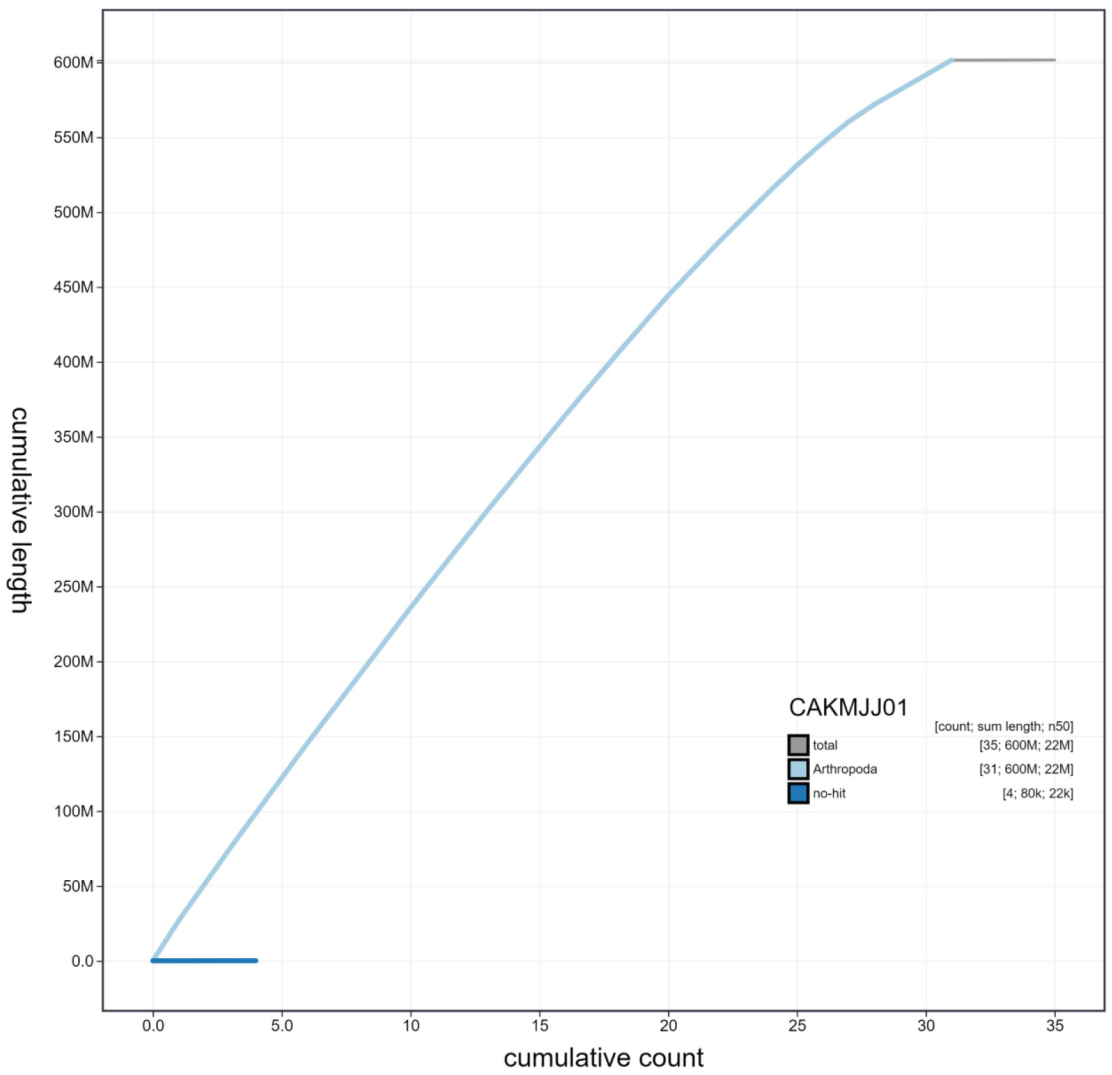
Genome assembly of *Luperina testacea*, ilLupTest1.1: cumulative sequence. BlobToolKit cumulative sequence plot. The grey line shows cumulative length for all scaffolds. Coloured lines show cumulative lengths of scaffolds assigned to each phylum using the buscogenes taxrule. An interactive version of this figure is available at https://blobtoolkit.genomehubs.org/view/ilLupTest1.1/dataset/CAKMJJ01/cumulative.

**Figure 5.**
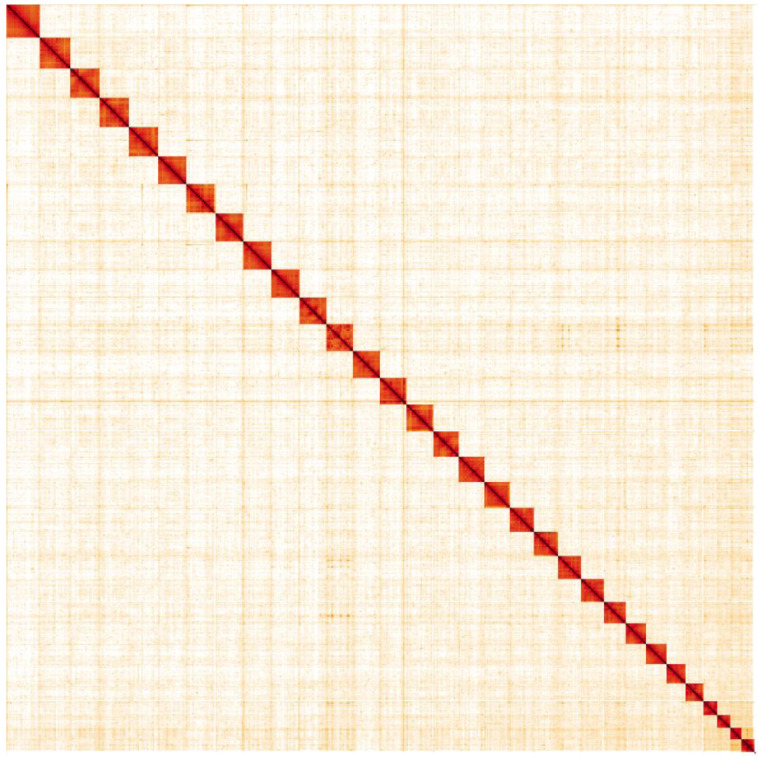
Genome assembly of *Luperina testacea*, ilLupTest1.1: Hi-C contact map. Hi-C contact map of the ilNotZicz1.1 assembly, visualised in HiGlass. Chromosomes are shown in order of size from left to right and top to bottom. An interactive version of this map is available here.

**Table 2.** Chromosomal pseudomolecules in the genome assembly of *Luperina testacea*, ilLupTest1.1.

INSDC accession	Chromosome	Size (Mb)	GC%
OV656756.1	1	24.71	37.8
OV656757.1	2	23.72	38.0
OV656758.1	3	23.38	37.9
OV656759.1	4	23.35	37.7
OV656760.1	5	23.31	38.0
OV656761.1	6	22.77	37.6
OV656762.1	7	22.63	37.6
OV656763.1	8	22.60	38.0
OV656764.1	9	22.54	37.7
OV656765.1	10	21.93	37.6
OV656766.1	11	21.69	38.1
OV656767.1	12	21.61	37.6
OV656768.1	13	21.13	37.8
OV656769.1	14	20.98	38.0
OV656770.1	15	20.97	37.8
OV656771.1	16	20.67	37.7
OV656772.1	17	20.09	38.1
OV656773.1	18	19.70	38.2
OV656774.1	19	19.66	37.9
OV656775.1	20	18.24	37.9
OV656776.1	21	18.03	38.1
OV656777.1	22	17.37	38.4
OV656778.1	23	17.07	38.1
OV656779.1	24	16.30	38.2
OV656780.1	25	15.06	38.2
OV656781.1	26	13.89	38.2
OV656782.1	27	11.55	38.9
OV656783.1	28	10.17	39.0
OV656784.1	29	9.84	39.0
OV656785.1	30	9.66	39.8
OV656755.1	Z	26.81	37.8
OV656786.1	MT	0.02	20.1
-	Unplaced	0.06	39.8

## Methods

### Sample acquisition and DNA extraction

A single male *L. testacea* (ilLupTest1) was collected from Hever Castle, England, UK (latitude 51.1884, longitude 0.1198) by Gavin Broad, Natural History Museum, using a light trap in grassland near a lake. The sample was identified by the same individual, and preserved in liquid nitrogen.

DNA was extracted at the Tree of Life laboratory, Wellcome Sanger Institute. The ilLupTest1 sample was weighed and dissected on dry ice with tissue set aside for Hi-C sequencing. Thorax tissue was cryogenically disrupted to a fine powder using a Covaris cryoPREP Automated Dry Pulveriser, receiving multiple impacts. Fragment size analysis of 0.01–0.5 ng of DNA was then performed using an Agilent FemtoPulse. High molecular weight (HMW) DNA was extracted using the Qiagen MagAttract HMW DNA extraction kit. Low molecular weight DNA was removed from a 200-ng aliquot of extracted DNA using 0.8X AMpure XP purification kit prior to 10X Chromium sequencing; a minimum of 50 ng DNA was submitted for 10X sequencing. HMW DNA was sheared into an average fragment size between 12–20 kb in a Megaruptor 3 system with speed setting 30. Sheared DNA was purified by solid-phase reversible immobilisation using AMPure PB beads with a 1.8X ratio of beads to sample to remove the shorter fragments and concentrate the DNA sample. The concentration of the sheared and purified DNA was assessed using a Nanodrop spectrophotometer and Qubit Fluorometer and Qubit dsDNA High Sensitivity Assay kit. Fragment size distribution was evaluated by running the sample on the FemtoPulse system.

### Sequencing

Pacific Biosciences HiFi circular consensus and 10X Genomics Chromium read cloud sequencing libraries were constructed according to the manufacturers’ instructions. Sequencing was performed by the Scientific Operations core at the Wellcome Sanger Institute on Pacific Biosciences SEQUEL II (HiFi) and Illumina NovaSeq 6000 (10X) instruments. Hi-C data were generated from head tissue using the Arima Hi-C+ kit and sequenced on NovaSeq 6000.

### Genome assembly

Assembly was carried out with Hifiasm ( Cheng *et al.*, 2021); haplotypic duplication was identified and removed with purge_dups ( Guan *et al.*, 2020). One round of polishing was performed by aligning 10X Genomics read data to the assembly with longranger align, calling variants with freebayes ( Garrison & Marth, 2012). The assembly was then scaffolded with Hi-C data ( Rao *et al.*, 2014) using SALSA2 ( Ghurye *et al.*, 2019). The assembly was checked for contamination as described previously ( Howe *et al.*, 2021). Manual curation ( Howe *et al.*, 2021) was performed using HiGlass ( Kerpedjiev *et al.*, 2018) and Pretext. The mitochondrial genome was assembled using MitoHiFi ( Uliano-Silva *et al.*, 2021), which performs annotation using MitoFinder ( Allio *et al.*, 2020). The genome was analysed and BUSCO scores generated within the BlobToolKit environment ( Challis *et al.*, 2020). Table 3 contains a list of all software tool versions used, where appropriate.

**Table 3.** Software tools used.

Software tool	Version	Source
Hifiasm	0.15.3	Cheng *et al.*, 2021
purge_dups	1.2.3	Guan *et al.*, 2020
SALSA	2.2	Ghurye *et al.*, 2019
longranger align	2.2.2	https://support.10xgenomics.com/genome-exome/software/pipelines/ latest/advanced/other-pipelines
freebayes	1.3.1-17- gaa2ace8	Garrison & Marth, 2012
MitoHiFi	2.0	Uliano-Silva *et al.*, 2021
HiGlass	1.11.6	Kerpedjiev *et al.*, 2018
PretextView	0.2.x	https://github.com/wtsi-hpag/PretextView
BlobToolKit	3.0.5	Challis *et al.*, 2020

### Ethics/compliance issues

The materials that have contributed to this genome note have been supplied by a Darwin Tree of Life Partner. The submission of materials by a Darwin Tree of Life Partner is subject to the Darwin Tree of Life Project Sampling Code of Practice. By agreeing with and signing up to the Sampling Code of Practice, the Darwin Tree of Life Partner agrees they will meet the legal and ethical requirements and standards set out within this document in respect of all samples acquired for, and supplied to, the Darwin Tree of Life Project. Each transfer of samples is further undertaken according to a Research Collaboration Agreement or Material Transfer Agreement entered into by the Darwin Tree of Life Partner, Genome Research Limited (operating as the Wellcome Sanger Institute), and in some circumstances other Darwin Tree of Life collaborators.

## Data availability

European Nucleotide Archive: Luperina testacea. Accession number PRJEB48331; https://identifiers.org/ena.embl/PRJEB48331.

The genome sequence is released openly for reuse. The *L. testacea* genome sequencing initiative is part of the Darwin Tree of Life (DToL) project. All raw sequence data and the assembly have been deposited in INSDC databases. The genome will be annotated and presented through the Ensembl pipeline at the European Bioinformatics Institute. Raw data and assembly accession identifiers are reported in Table 1.

## Author information

Members of the Natural History Museum Genome Acquisition Lab are listed here: https://doi.org/10.5281/zenodo.5746819.

Members of the Darwin Tree of Life Barcoding collective are listed here: https://doi.org/10.5281/zenodo.5744972.

Members of the Wellcome Sanger Institute Tree of Life programme are listed here: https://doi.org/10.5281/zenodo.6125027.

Members of Wellcome Sanger Institute Scientific Operations: DNA Pipelines collective are listed here: https://doi.org/10.5281/zenodo.5746904.

Members of the Tree of Life Core Informatics collective are listed here: https://doi.org/10.5281/zenodo.6125046.

Members of the Darwin Tree of Life Consortium are listed here: https://doi.org/10.5281/zenodo.5638618.
