# Supplementary material for: The genome sequence of the flounced rustic, Luperina testacea (Denis & Schiffermüller, 1775)
Source: Wellcome Open Res. 2022 Apr 5;7:127. [Version 1] doi: 10.12688/wellcomeopenres.17816.1 (PMC11362736; doi:10.12688/wellcomeopenres.17816.1)
Supplement: Peer review history

# Reviewer report: Reviewer response for version 1

**DOI:** 10.21956/wellcomeopenres.19724.r69869

**Published:** 2024-08-29

**Authors:** Nicholas W VanKuren

**Affiliations:** 1 Department of Ecology & Evolution, The University of Chicago, Chicago, Illinois, USA

Broad and co-authors present a complete assembly of a common British moth, *Luperina testacea*. They use the standard pipeline for genome sequencing and assembly that have been applied to hundreds of TOL genomes over the past several years. 

 I sincerely appreciate the high quality and accessible data from all of these genome assembly projects, but I am frequently lost as to *why* we need to sequence to them all beyond "we can". I would love to see a very brief discussion of why this moth is particularly interesting, or at least into what context this genome sequence is being placed (does it fill some crucial phylogenetic gap, is it a pest, does it have a unique ecology?) It doesn't need to be regurgitated from any of the previous TOL/Sanger pubs, but just a sentence or two to give me a reason for why I should look at this genome would help a lot. 

 I have no issues with any of the methods, the excellent tools to access and analyze the data, or the sequence itself.

Are sufficient details of methods and materials provided to allow replication by others?

Yes

Is the rationale for creating the dataset(s) clearly described?

No

Are the datasets clearly presented in a useable and accessible format?

Yes

Are the protocols appropriate and is the work technically sound?

Yes

Reviewer Expertise:

genomics, genetics, phylogenetics, development

I confirm that I have read this submission and believe that I have an appropriate level of expertise to confirm that it is of an acceptable scientific standard.

# Reviewer report: Reviewer response for version 1

**DOI:** 10.21956/wellcomeopenres.19724.r69876

**Published:** 2023-11-30

**Authors:** Francesco Cicconardi

**Affiliations:** 1 Department of Zoology, University of Cambridge & School of Biological Sciences, University of Bristol, Bristol, England, UK

The genome assembly of *Luperina testacea *is reported here.

 As usual for the genomes from the tree of life, they are high-quality data, using a combination of long and linked reads. My only concern is related to the method section which is not very detailed and a carbon copy of other papers. A bit disappointing to me.

Are sufficient details of methods and materials provided to allow replication by others?

No

Is the rationale for creating the dataset(s) clearly described?

Yes

Are the datasets clearly presented in a useable and accessible format?

Yes

Are the protocols appropriate and is the work technically sound?

Yes

Reviewer Expertise:

Genomics

I confirm that I have read this submission and believe that I have an appropriate level of expertise to confirm that it is of an acceptable scientific standard, however I have significant reservations, as outlined above.

# Reviewer report: Reviewer response for version 1

**DOI:** 10.21956/wellcomeopenres.19724.r52298

**Published:** 2022-09-27

**Authors:** Susan McEvoy

**Affiliations:** 1 EEB, Meyer Lab, University of California Santa Cruz, Santa Cruz, CA, USA; 2 Santa Barbara Botanic Garden, Mission Canyon, CA, USA

This article details the genome assembly of *Luperina testacea*, the flounced rustic moth of northwest and central Europe. Tissue from the thorax of a single male *L. testacea* was used for Pacific Biosciences HiFi and Chromium 10X sequencing, while the head was used to generate Arima Hi-C data. The HiFi reads were assembled with HiFiasm, polished with 10X reads in longranger, and scaffolded with Hi-C reads in SALSA2. The mitochondrial genome was assembled using MitoHiFi. The genome was manually curated using a visualization of the assembly in HiGlass. The final reference was highly complete and contiguous relative to standard genome reference metrics, with 31 pseudomolecules and a sex chromosome, totaling 601 Mbp in length, and with 98.9% of BUSCO Lepidoptera genes found in a single copy.

 Standard genome assembly protocols were used, and this article communicates details in a reproducible manner. The resulting datasets are also very clear. Any comments I can contribute are very minor and mostly just things I was curious about.

 I marked “partial” regarding the rationale for creating the dataset just because it didn’t seem explicit. My assumption is that it is because it is common across Europe as mentioned in the background. How does this genome fit into the current landscape of Lepidoptera genomics? Or maybe a similar statement? Also, as a plant specialist, I wasn’t sure of the phrase “regular at light”. If it is specific to entomology, perhaps it could be rewritten to be clear to a broader audience. 

 The protocols as described for DNA extraction, sequencing, assembly, and scaffolding are generally appropriate and commonly used elsewhere. I was a little puzzled at polishing the assembly with 75x of Chromium 10X instead of a whole-genome shotgun. 10X is normally used for scaffolding, but perhaps it was not needed for this purpose as the Hi-C scaffolding was sufficient. I believe 10X would have a coverage bias that makes it less than ideal for polishing genome-wide, but maybe at 75x it is fine. I don’t think this is a step I would recommend others replicate. 

 I was also wondering if there are any potential issues with separating thorax and head tissue and only using head for Hi-C. I'm just curious if there would be any issues with this. Maybe it is common with insects, I'm not sure.

Are sufficient details of methods and materials provided to allow replication by others?

Yes

Is the rationale for creating the dataset(s) clearly described?

Partly

Are the datasets clearly presented in a useable and accessible format?

Yes

Are the protocols appropriate and is the work technically sound?

Yes

Reviewer Expertise:

Plant genomics: assembly, annotation, comparative genomics, transcriptomics, methylomics, conservation genomics.

I confirm that I have read this submission and believe that I have an appropriate level of expertise to confirm that it is of an acceptable scientific standard.
